# The Transcription Factor Pou3f1 Sheds Light on the Development and Molecular Diversity of Glutamatergic Cerebellar Nuclear Neurons in the Mouse

**DOI:** 10.3389/fnmol.2022.921901

**Published:** 2022-07-20

**Authors:** Joshua Po Han Wu, Joanna Yeung, Maryam Rahimi-Balaei, Sih-Rong Wu, Huda Zoghbi, Dan Goldowitz

**Affiliations:** ^1^Department of Medical Genetics, Centre for Molecular Medicine and Therapeutics, BC Children’s Hospital Research Institute, University of British Columbia, Vancouver, BC, Canada; ^2^Jan and Dan Duncan Neurological Research Institute at Texas Children’s Hospital, Houston, TX, United States; ^3^Department of Neuroscience, Baylor College of Medicine, Houston, TX, United States; ^4^Department of Molecular and Human Genetics, Baylor College of Medicine, Houston, TX, United States; ^5^Howard Hughes Medical Institute, Baylor College of Medicine, Houston, TX, United States

**Keywords:** mouse, development, cerebellum, Atoh1, Pax6, Tbr1, Brn2, Irx3

## Abstract

The cerebellar nuclear (CN) neurons are a molecularly heterogeneous population whose specification into the different cerebellar nuclei is defined by the expression of varying sets of transcription factors. Here, we present a novel molecular marker, Pou3f1, that delineates specific sets of glutamatergic CN neurons. The glutamatergic identity of Pou3f1^+^ cells was confirmed by: (1) the co-expression of vGluT2, a cell marker of glutamatergic neurons; (2) the lack of co-expression between Pou3f1 and GAD67, a marker of GABAergic neurons; (3) the co-expression of Atoh1, the master regulator required for the production of all cerebellar glutamatergic lineages; and (4) the absence of Pou3f1-expressing cells in the *Atoh1*-null cerebellum. Furthermore, the lack of Pax6 and Tbr1 expression in Pou3f1^+^ cells reveals that Pou3f1-expressing CN neurons specifically settle in the interposed and dentate nuclei. In addition, the Pou3f1-labeled glutamatergic CN neurons can be further classified by the expression of Brn2 and Irx3. The results of the present study align with previous findings highlighting that the survival of the interposed and dentate CN neurons is largely independent of Pax6. More importantly, the present study extends the field’s collective knowledge of the molecular diversity of cerebellar nuclei.

## Introduction

The cerebellar nuclei are the primary outputs from the cerebellar cortex to the rest of the brain. Therefore, they constitute a very critical structure within the cerebellum. However, despite our knowledge that each nucleus projects to distinct brain regions (Ruigrok et al., 2015), how the CN neurons get specified and settle into the different nuclei, remain relatively unexplored.

Neurogenesis of cerebellar nuclei begins early during cerebellar development. In mice, it begins as early as embryonic (E) day 10. The neurons that populate these nuclei can be classified based on the neurotransmitter they secrete, with those secreting glutamate (glutamatergic) or GABA (GABAergic) being the predominant populations. Glutamatergic CN neurons are known to arise from the Atoh1-expressing rhombic lip (RL; Machold and Fishell, 2005; Wang et al., 2005). As they leave the RL, they migrate along the subpial stream (SPS), then temporarily accumulate in a dorsal region called the nuclear transitory zone (NTZ) at E15, before descending into the cerebellar white matter (Altman and Bayer, 1997). In addition to the importance of Atoh1 in the neurogenesis of glutamatergic CN neurons, Pax6 has also been demonstrated to play a role in regulating the survival of these neurons (Yeung et al., 2016). However, as there are three cerebellar nuclei in the mouse cerebellum (fastigial/medial, interposed, and dentate/lateral), Pax6’s influence in the development of glutamatergic CN neurons seems to be most pronounced in those populating the fastigial nucleus (Fink et al., 2006; Yeung et al., 2016), suggesting that there are other important molecular players governing the development of the interposed and dentate CN neurons. It was commented in an excellent review of CN development in 2013 that, in general, “very little is known about the presence of topographical molecular maps within the four subdivisions of the CN” (Elsen et al., 2012); and not that much has been added to the story a decade later.

Seeking to characterize the molecular diversity of CN neurons, a recent study by Kebschull et al. (2020) performed single-nucleus RNA sequencing on adult mouse cerebella. The results revealed that RL-derived excitatory CN neurons can be categorized into as many as 15 distinct subtypes, with most diversity located in the interposed and dentate nuclei. However, we find that few, if any, of the molecules important for glutamatergic CN neuron development are expressed in the adult cerebellum, suggesting that the molecules that provide the adult CN blueprint do not mirror those involved in the developmental period. This discrepancy thus prompted us to explore molecules that are specifically expressed in glutamatergic CN neurons during development; with a particular emphasis on those eventually settling in the interposed and dentate nuclei.

## Materials and Methods

### Mouse Strains and Husbandry

C57BL/6J mice were used for the characterization of wild-type (WT) Pou3f1 expression pattern during development.

The *Pax6* mutant strain, *Pax6^Sey^* (originally obtained from Robert Grainger and Marilyn Fisher, University of Virginia), was bred as heterozygous pairs, phenotyped for eye size and presence of cataracts, and genotyped as previously described (Swanson et al., 2005). *Pax6^Sey^*^/*Sey*^ embryos were generated by intercrossing *Pax6^Sey/+^* mice.

The *Atoh1-lacZ* reporter strain, Atoh1^β-Gal^ (obtained from Huda Zoghbi, Baylor College of Medicine), was genotyped by PCR according to the protocol previously described (Jensen et al., 2002). *Atoh1*-null embryos, *Atoh1*^β-Gal/β-Gal^, were generated by intercrossing *Atoh1^β-Gal/+^* mice.

All embryonic ages utilized in these experiments were dated from the appearance of a vaginal plug. The morning that a vaginal plug was detected was designated as embryonic day (E) 0.5.

Breeding and colony maintenance were performed in the pathogen-free mouse core facility of the Centre for Molecular Medicine and Therapeutics at the University of British Columbia (UBC). All mice were housed under the following conditions: 14-h light/10-h dark cycle, 20 ± 2°C with 50 ± 5% relative humidity, as well as food and water *ad libitum*. Studies were conducted according to the protocols approved by the Institutional Animal Care and Use Committee and the Canadian Council on Animal Care at UBC.

### Tissue Preparation and Histology

Embryos were collected every day from E10.5 to E18.5. Neonatal pups were collected at P0 and P6. Embryos harvested between E10.5 to E15.5 were fixed by immersion in 4% paraformaldehyde in 0.1 M phosphate buffer (PB, pH 7.4) for 1 h at 4°C. Embryos or pups harvested at E16.5 and later were transcardially perfused first with phosphate buffered saline (PBS), and then with 4% paraformaldehyde in 0.1 M PB. Brains were isolated and further fixed in 4% paraformaldehyde in 0.1 M PB for one h at room temperature. Fixed tissues were rinsed with PBS, followed by cryoprotection with 30% sucrose/PBS overnight at 4°C before embedding in the Optimal Cutting Temperature (O.C.T.) compound (4583, Sakura Finetek, USA). Tissues were sectioned in a cryostat at 12 μm thickness for immunofluorescence (IF) and *in situ* hybridization (ISH). Cryosections were mounted on Superfrost^TM^ slides (12-500-15, Fisher Scientific), air dried at room temperature, and stored at −80°C until used. Sagittal sections were cut from one side of the cerebellum to the other (left to right, or vice versa). Coronal sections were cut from the front of the cerebellum to the back. In all cases, observations were based on a minimum of three embryos per genotype per experiment.

### *In situ* Hybridization

All ISH experiments were performed on tissues prepared as described above. Sense and antisense riboprobes corresponding to the cDNA fragment were synthesized and labeled with digoxygenin (DIG)-UTP. A cDNA library was obtained from an E15.5 mouse brain using a cDNA synthesis kit (K1681, Thermo Scientific). Then the corresponding cDNA of *Pou3f1* was produced with this cDNA library, using the gene-specific forward and reverse primers (forward: 5’-AGCAGCGGAAGATCCAGAAT-3’; reverse: 5’-TCGGTTTAGTCGGGCATACA-3’). These primers flank the 3’ untranslated region and yield a product of 1,088 bp; both the targeting region and length of the probe are important factors contributing to higher ISH specificity. Each of the resultant cDNAs was cloned into the pGEM-T Easy vector (A1360, Promega) for the generation of cDNA templates. cDNA templates for the sense and antisense riboprobes were specifically made using the primers M13F: 5’-GTTTTCCCAGTCACGAC-3’ or M13R: 5’-CAGGAAACAGCTATGAC-3’ and the *Pou3f1*-specific forward or reverse primers. Riboprobes were produced using the SP6 or T7 RNA polymerase (#EP0133 and #EP0111, Thermo Scientific, respectively) with the corresponding cDNA templates. The resultant riboprobes were precipitated using 5 M ammonium acetate and 100% EtOH in an RNase-free environment. Riboprobes were denatured at 72°C for 10 min and incubated on ice for 5 min, then mixed with ULTRAhyb hybridization buffer (AM8670, Applied Biosystems) preheated at 68°C. Prior to hybridization, sections were acetylated with acetic anhydride in 0.1 M triethanolamine at pH 8.0 and dehydrated with graded concentrations of RNAse-free ethanol. Sections were first incubated with ULTRAhyb hybridization buffer at 68°C in a humid chamber for 1.5 h, then replaced with riboprobe in ULTRAhyb hybridization buffer at 68°C overnight. After hybridization, the slides were rinsed with descending concentrations of salt: 4× SSC, 50% formamide in 2× SSC, and 2× SSC at 55°C. Sections were then treated with RNAse A in 2× SSC at 37°C for 30 min, followed by 2× SSC, 1× SSC, and 0.5× SSC at 55°C. Afterward, the sections were incubated with an anti-Dig antibody (11093274910, Roche) for 2 h at room temperature. The slides were washed with maleic buffer, followed by reaction buffer, then the slides were colorized with NBT/BICP (11681451001, Roche). The reaction buffer was prepared according to the manufacturer’s manual of NTP/BICP (11681451001, Roche). Following colorization, the slides were rinsed with 0.1 M PB, then post-fixed in 4% paraformaldehyde, and washed with distilled water. The slides were dehydrated with graded concentrations of ethanol and xylenes. In the end, cover slips were applied to the slide, using Permount (SP15-500, Fisher Scientific) as the mounting medium.

### Immunofluorescence

For immunofluorescence, tissue sections were first rehydrated in PBS three times, 5 min each, followed by a phosphate buffered saline with Triton X-100 (PBS-T) wash for 5 min (Fisher Scientific, BP 151). Sections were then incubated at room temperature for 1 h with a blocking solution (0.3% BSA, 10% normal goat serum, 0.02% sodium azide in PBS-T). Following the blocking step, the slides were subsequently incubated with the primary antibody in incubation buffer (0.3% BSA, 5% normal goat serum, 0.02% sodium azide in PBS-T) at room temperature overnight in a humid chamber. Primary antibodies used were as follows: rabbit anti-Atoh1 (1:500; Proteintech, 21215-1 AP), rabbit anti-Brn2 (1:1,000; Abcam, Ab94977), rabbit anti-GAD67 (1:500; ThermoFisher, PA5-21397), rabbit anti-Irx3 (1:8,000; a gift from Tom Jessell, Columbia University), rabbit anti-Pax6 (1:200; Covance, PRB-278P, RRID:AB_291612), mouse anti-Pou3f1 (1:500; Millipore, MABN738), rabbit anti-Tbr1 (1:800; Abcam, Ab31940, RRID:AB_2200219), and chicken anti-vGluT2 (1:500; Synaptic Systems, 135 416). Following the overnight incubation, the slides were immersed in three, 10-min PBS-T rinses. The sections were then incubated with the corresponding secondary antibodies at room temperature for 1 h, followed by three 0.1 M PB washes and one 0.01 M PB wash. The secondary antibodies used included: goat anti-mouse 488 (1:500; Fisher Scientific; A-11001), goat anti-mouse 594 (1:500; Fisher Scientific; A-11020), goat anti-rabbit 488 (1:500; Fisher Scientific; A-11008), and goat anti-chicken 488 (1:500; Fisher Scientific; A-11039). Coverslips were applied to the slides using the FluorSave mounting medium (Cat #: 345789, Calbiochem).

### Microscopy

Analysis and photomicroscopy were performed using a Zeiss Axiovert 200 M microscope with Axiocam/Axiovision hardware-software components (Carl Zeiss).

Confocal microscopy was performed using a Leica SP8X STED white light laser confocal microscope with Leica Application Suite X software (Leica Microsystems). Images were taken with the 20× objective lens (HC PL APO 20×/0.75 CS2, WD 0.62 mm). Diode laser of 405 nm was used to image the fluorescent dye DAPI, and white light laser (power at 50%) was used to image Alexa Fluor 488 and Alexa Fluor 594. Signals were detected with HyD detectors with the following configurations (see Table below).

**Table:** 

**Dye**	**LUT**	**Laser line**	**AOTF**	**Detector**	**Gain**	**Detection range**	**Time gating**
DAPI	Blue	405 nm	1.9984%	HyD	100%	410–513 nm	NA
Alexa 488	Green	499 nm	15.0007%	HyD	100%	504–591 nm	0.3–6 ns
Cy3	Red	598 nm	7.0015%	HyD	100%	603–765 nm	0.3–6 ns

### Analysis and Quantification

We estimated the average total number of CN neurons per E18.5 embryo positive for the corresponding cell marker—Pou3f1, Brn2, or Irx3—by counting the number of positive cells in coronal sections that spanned the cerebellar nuclear region with sampling made at 10-section intervals across that region. The count from each section was multiplied by 10 to account for sampling, then summed to arrive at an estimated total number of labeled cells.

In all cases, observations were based on a minimum of three cerebella per genotype. Standardization of intensity, as well as cell counting, were performed using ImageJ’s built-in functions. Statistical significance in the difference of averaged Pou3f1^+^ cell numbers among the WT, *Sey*/+, and *Sey/Sey* cerebella was determined by One-Way ANOVA with *post-hoc* test.

## Results

### The Early Expression Pattern of Pou3f1 in the Developing Cerebellum

**Figure 1:**
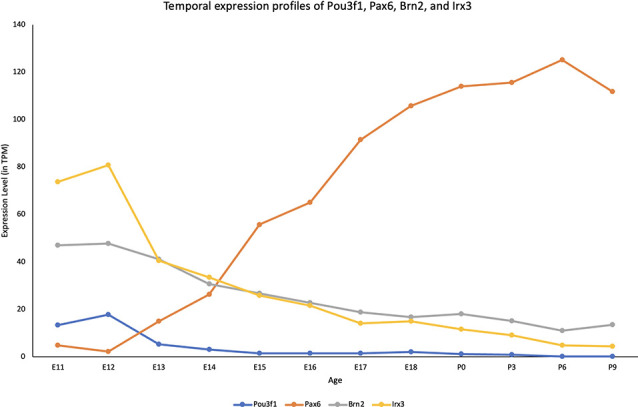
The transcriptional profile of *Pou3f1* during cerebellar development based upon the RIKEN FANTOM5 data. The *y*-axis shows the expression level in transcripts per million (TPM). The *x*-axis represents developmental timepoints, from embryonic (E) to postnatal (P) ages.

To quantitatively determine the expression profile of Pou3f1 across developmental timepoints, we examined the cerebellar transcriptomic dataset generated in collaboration with the FANTOM5 Consortium that spanned both embryonic and neonatal (P) timepoints (E11-P9; Arner et al., 2015; Ha et al., 2019). The dataset revealed that within the cerebellum, Pou3f1 expression peaks at E12, then gradually decreases as development progresses (Figure 1). To provide more insight into the temporal expression profile of *Pou3f1*, we compared the *Pou3f1* expression level to those of other known CN neurons markers, *Pax6, Brn2, and Irx3* (Figure 1). Notably, *Pou3f1* and *Pax6* expression demonstrate contrasting patterns, whereby *Pou3f1* expression decreases with time, while *Pax6* expression increases with time. On the other hand, the expression profile of *Pou3f1* parallels those of *Brn2* and *Irx3* (Figure 1).

**Figure 2:**
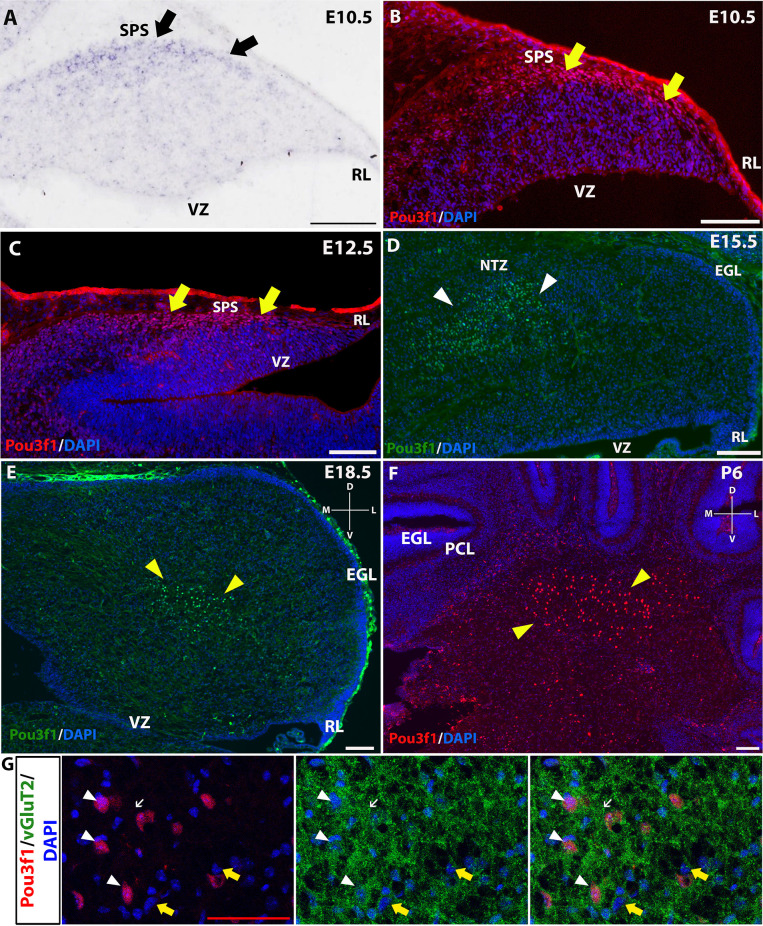
The expression pattern of Pou3f1 across space and time. **(A–C)** In the E10.5 and E12.5 cerebella, *in situ* hybridization (ISH) **(A)** and immunofluorescent (IF) staining **(B,C)** reveal that Pou3f1 is expressed in cells along the subpial stream (SPS, arrows). **(D)** In the E15.5 cerebellum, Pou3f1 is expressed primarily in the nuclear transitory zone (NTZ, white arrowheads). **(E,F)** By E18.5, Pou3f1-expressing cells have reached the cerebellar white matter (yellow arrowheads), and continue to populate the cerebellar white matter at P6 (yellow arrowheads). **(G)** In the P6 cerebellum, Pou3f1^+^ cells co-express vGluT2 (white arrowheads). vGluT2 proteins in the cerebellar white matter are localized to the cell bodies (white arrowheads) and the synaptic boutons (white arrows). vGluT2^−^ cells are also found in the cerebellar white matter (yellow arrows). Panels **(A–D)** show sagittal sections of the cerebellum, while sections shown in **(E–G)** are in the coronal plane. D, dorsal; EGL, external germinal layer; L, lateral; M, medial; NTZ, nuclear transitory zone; PCL, Purkinje cell layer; RL, rhombic lip; SPS, subpial stream; V, ventral; VZ, ventricular zone. White scale bars, 100 μm. Red scale bar, 50 μm.

**Figure 3:**
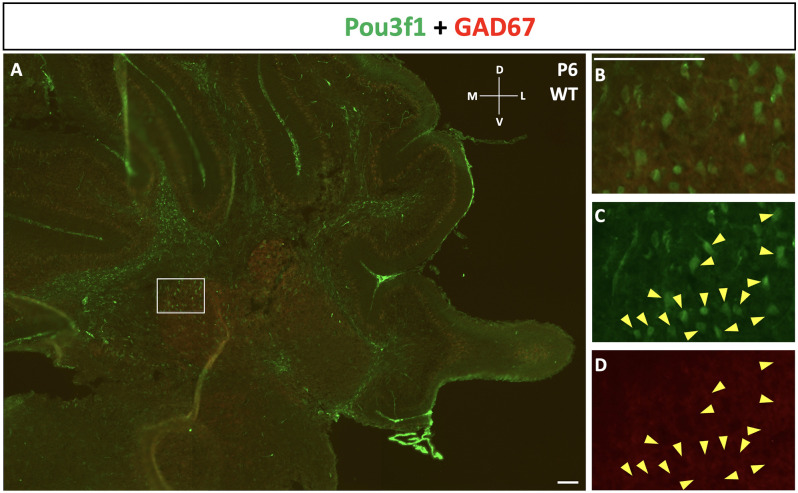
The lack of co-expression between Pou3f1 (green) and GAD67 (red). **(A)** A representative P6 coronal cerebellar section showing the relative expression patterns of Pou3f1 and GAD67, and the absence of co-expression between the two molecules. **(B–D)** Within the cerebellar white matter, Pou3f1^+^ cells are not surrounded by GAD67 puncta, demonstrating that they are not GABAergic. Panel **(B)** shows merged green and red channels, while **(C)** and **(D)** show individual channels. D, dorsal; L, lateral; M, medial; V, ventral. Scale bars, 100 μm.

To complement the quantitative understanding of Pou3f1 expression, we performed *in situ* hybridization (ISH) and immunofluorescence (IF) to obtain the spatial profile of the Pou3f1 mRNA transcript and protein. At E10.5, ISH revealed that the *Pou3f1* mRNA transcript is expressed prominently along the subpial stream (SPS), but not in the ventricular zone (VZ; Figure 2A). The IF analysis at E10.5 demonstrated the same expression pattern as ISH in the SPS (Figure 2B). Between E12.5 and E15.5, IF highlighted that Pou3f1^+^ cells begin to accumulate in the nuclear transitory zone (NTZ) and nowhere else in the developing cerebellum (Figures 2C,D). By E18.5, Pou3f1-labeled cells cluster in the forming cerebellar nuclear region, with no noticeable change in localization postnatally at P6 (Figures 2E,F). It is important to note that while Pou3f1 remains prominently expressed in the CN region across developmental timepoints, the decline in the transcriptomic profile shown in Figure 1 is attributable to the fact from a whole-tissue standpoint, the ratio of Pou3f1^+^ cells to the total number of cerebellar cells declines over time due to the proliferation of granule cells. Altogether, the early expression pattern of Pou3f1 along the SPS and its absence in the VZ suggest that Pou3f1-expressing cells are glutamatergic CN neurons. To test this hypothesis, we examined the relative expression patterns of Pou3f1 and vGluT2, a marker of glutamatergic neurons, using IF (Figure 2G). Indeed, at P6, Pou3f1-labeled cells co-express vGluT2; Pou3f1 exhibits nuclear expression, while vGluT2 exhibits cytoplasmic expression in the same cells. On the other hand, when the expression of Pou3f1 and GAD67, a GABAergic cell marker, was examined using IF (Figure 3A), none of the Pou3f1-labeled cells is surrounded by GAD67 puncta (Figures 3B–D).

### Pou3f1^+^ Cells Are Derived From Atoh1-Expressing Progenitors

**Figure 4:**
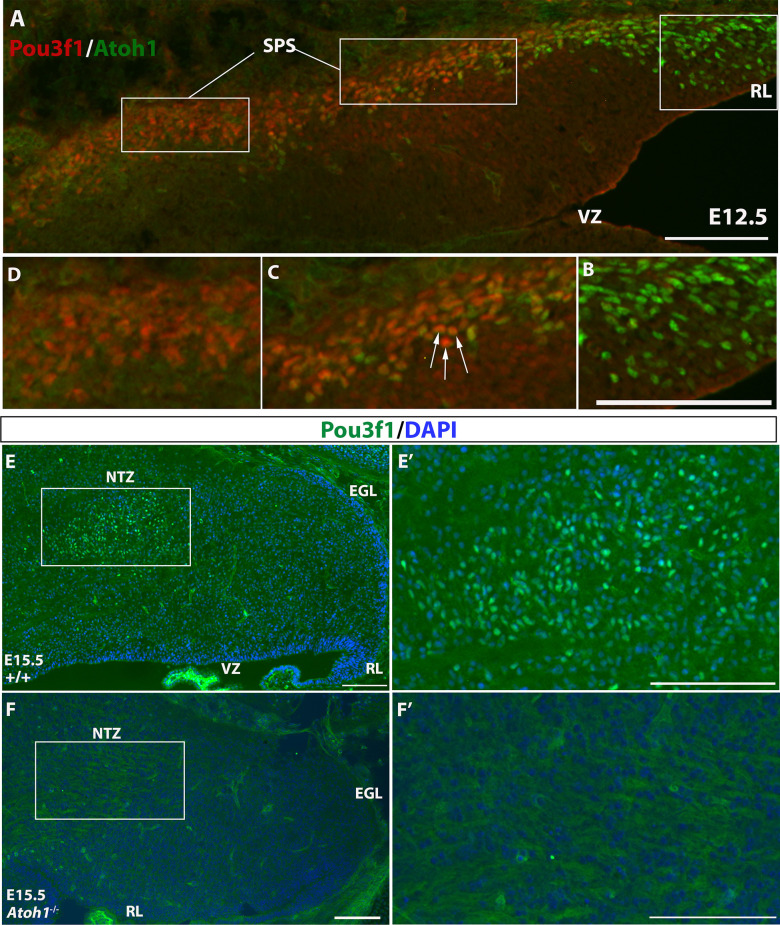
The expression patterns of Atoh1 and Pou3f1 in the wild-type and *Atoh1*-null cerebella. **(A)** Three molecular regions are demarcated by the expression of Atoh1 and Pou3f1 in the E12.5 cerebellar anlage (sagittal view): the rhombic lip (RL) is exclusively Atoh1^+^ (**B, right**), followed by the beginning portion of the subpial stream (SPS) characterized by co-localization of the two transcription factors (**C, middle**, white arrows), and the rest of the SPS marked exclusively by Pou3f1 (**D, left**). **(E,E’)** In the E15.5 wild-type cerebellum (sagittal view), Pou3f1 expression is present in the nuclear transitory zone (NTZ). The boxed area in **(E)** is shown in **(E’)** in higher magnification. **(F,F’)** However, in the corresponding *Atoh1*-null mutant cerebellum, Pou3f1^+^ cells are completely absent. The boxed area in **(F)** is shown in **(F’)** in higher magnification. EGL, external germinal layer; NTZ, nuclear transitory zone; RL, rhombic lip; SPS, subpial stream; VZ, ventricular zone. Scale bars, 100 μm.

To further verify the glutamatergic nature of Pou3f1^+^ cells, we examined their relationship to the Atoh1^+^, the definitive marker of glutamatergic CN neurons during development that arise in the RL (Machold and Fishell, 2005; Wang et al., 2005). Double label experiments were conducted between Pou3f1 and Atoh1 using IF at an early embryonic stage when Atoh1^+^ cells just emerge from the RL and Pou3f1 expression is observed in the SPS. At E12.5, IF co-staining of Pou3f1 and Atoh1 demonstrates three distinct regions (Figure 4A): (1) Atoh1^+^/Pou3f1^−^ cells in the posterior and lateral part of the RL (Figure 4B); (2) Atoh1^+^/Pou3f1^+^ double-labeled cells in the part of the SPS proximal to the RL (Figure 4C); and (3) Atoh1^−^/Pou3f1^+^ cells in the more distal aspects of the SPS (Figure 4D). The second class of co-stained cells appears as a transitional zone between the RL and the SPS, revealing an interesting region in which cells are positive for both Pou3f1 and Atoh1. Quantification of the numbers of Atoh1^+^, Pou3f1^+^, and Atoh1^+^/Pou3f1^+^ double-labeled cells revealed that on average, there are 53 Atoh1^+^ cells per sagittal section (12 μm in thickness), 66 Pou3f1^+^ cells per section, and 25 double-labeled cells per section. Not only does the co-localization of Atoh1 and Pou3f1 suggest that Pou3f1^+^ neurons are of glutamatergic lineage, but also hints that Pou3f1^+^ cells emerge from the Atoh1-expressing counterparts in close relationship to the cessation of Atoh1 expression.

### Pou3f1 Expression Persists in the Absence of Pax6

**Figure 5:**
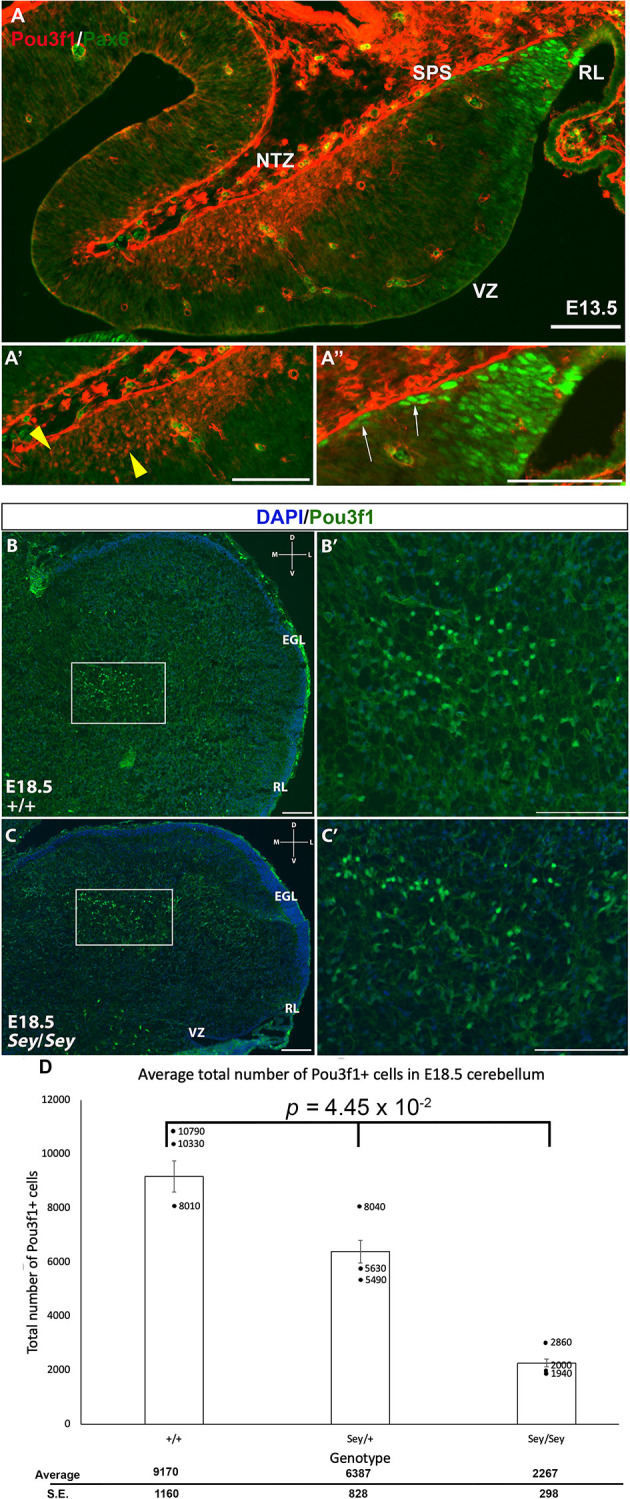
The absence of co-localization between Pou3f1 and Pax6 in the wild-type cerebellum and the persistence of Pou3f1 expression in the Small Eye (*Sey*) mutant cerebellum. **(A)** In the E13.5 cerebellum (sagittal view), lack of co-localization between Pou3f1 and Pax6 is evident, with Pou3f1-labeled cells predominantly in the nuclear transitory zone (NTZ; **A’**, yellow arrowheads), whereas Pax6-labeled cells are predominantly in the rhombic lip (RL) and the subpial stream (SPS; **A”**, white arrows). **(B,B’)** In the E18.5 wild-type cerebellum (coronal view), Pou3f1-expressing cells are found to reside in the cerebellar white matter. The boxed area in **(B)** is shown in **(B’)** in higher magnification. **(C,C’)** In the E18.5 *Sey* cerebellum (coronal view), Pou3f1-expressing cells are present. The boxed area in **(C)** is shown in **(C’)** in higher magnification. **(D)** One-Way ANOVA reveals that the average total number of Pou3f1^+^ cells in the E18.5 *Sey* coronal section is significantly different from those in the heterozygous mutant and wild-type counterparts (*p* = 4.45 × 10^−2^). Individual points represent the numbers from each animal counted. D, dorsal; EGL, external germinal layer; L, lateral; M, medial; NTZ, nuclear transitory zone; RL, rhombic lip; V, ventral; VZ, ventricular zone. Error bars represent SE. Scale bars, 100 μm.

Pax6 is known to act downstream of Atoh1, and is important for the development of RL-derived cell types, including granule cells, unipolar brush cells (UBCs), and glutamatergic CN neurons (Fink et al., 2006; Yeung et al., 2016). Pax6 is expressed in the glutamatergic CN neurons along the SPS, and its expression is downregulated as they enter the NTZ (Fink et al., 2006; Yeung et al., 2016). Thus, one expectation was that Pou3f1 and Pax6 are co-expressed in the cells in the SPS. To test this, we performed IF co-staining for Pou3f1 and Pax6 at E13.5 in the WT cerebella. Contrary to our expectation, the two proteins are not co-expressed at this timepoint (Figure 5A). Specifically, Pou3f1-expressing cells are localized to the NTZ (Figure 5A’), while Pax6-expressing cells are restricted to the RL and the beginning of the SPS (Figure 5A”). The absence of co-labeling between Pou3f1 and Pax6 at E13.5 was unexpected, as it was reported previously that Pax6 is critical for the development of glutamatergic CN neurons (Yeung et al., 2016).

The potential relationship between Atoh1 and Pou3f1 was further examined in mice with homozygous knockout of *Atoh1*. In *Atoh1*-null mice, glutamatergic CN neurons are eliminated (Wang et al., 2005). Therefore, because Pou3f1 appears to selectively delineate glutamatergic CN neurons, the expectation was that Pou3f1^+^ neurons would be absent in the *Atoh1*-null cerebellum. To test this hypothesis, we performed Pou3f1 IF staining on E15.5 WT and *Atoh1*-null cerebella. While in the E15.5 WT cerebellum Pou3f1 is expressed in cells of the NTZ (Figures 4E,E’), in the E15.5 *Atoh1*-null cerebellum Pou3f1-expressing cells are mostly absent (Figures 4F,F’). The residual Pou3f1 expression is attributable to the fact that in the *Atoh1^B-Gal/B-Gal^* animals, there are very few residual Atoh1-expressing cells (Jensen et al., 2004). On average, at E15.5, there are 72 Pou3f1^+^ cells per WT sagittal section, vs. six Pou3f1^+^ cells per *Atoh1*-null section. Altogether, an investigation of the spatiotemporal relationship between Pou3f1 and Atoh1 indicates that Pou3f1^+^ neurons are *Atoh1*-dependent, and are indeed glutamatergic CN neurons.

The lack of co-expression between Pou3f1 and Pax6 suggests an independent relationship and hints at the idea that Pou3f1-labeled CN neurons do not require Pax6 for their generation. To test this hypothesis, we examined the expression of Pou3f1 in the *Pax6*-null Small Eye (*Sey*) mutant. As can be seen in Figure 4; Pou3f1 expression persists in the *Sey* mutant (Figures 5B,B’,C,C’). At E18.5, Pou3f1^+^ cells are located in the cerebellar white matter, which parallels their expression pattern in the WT cerebella. To determine the quantitative relationship between *Pax6*- and *Pou3f1*-expressing cells, we calculated the average total number of Pou3f1^+^ cells in the WT, *Pax6*-heterozygous, and *Pax6-null* cerebella. Interestingly, at E18.5, a One-Way ANOVA reveals that the average total number of Pou3f1^+^ neurons in the *Sey* mutant cerebellum is significantly lower than those in the heterozygous mutant and WT cerebella (*p* = 4.45 × 10^−2^; Figure 5D). This finding thus points to a deeper relationship between Pou3f1 and Pax6, whereby at least a portion of the Pou3f1-expressing population requires Pax6 for their generation or specification.

### Pou3f1 Defines a Population of Glutamatergic CN Neurons Distinct From the Tbr1^+^ Population

**Figure 6:**
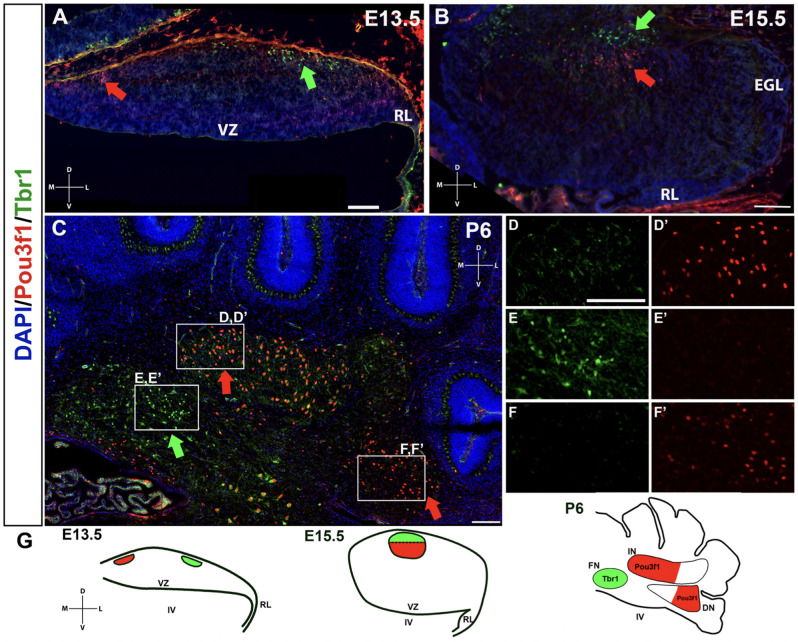
The non-overlapping expression patterns of Pou3f1 and Tbr1 throughout development. **(A)** In the E13.5 wild-type cerebellum (coronal view), Pou3f1-expressing cells are located more medially than Tbr1-expressing cells (red and green arrows, respectively). **(B)** By E15.5, as shown in the wild-type cerebellum (coronal view), Pou3f1^+^ CN neurons and Tbr1^+^ CN neurons are found in the same plane along the medial-lateral axis (red and green arrows, respectively). However, the Pou3f1^+^ CN neurons tend to cluster more ventrally compared to Tbr1^+^ population. **(C)** At P6, the cluster of Pou3f1-labeled CN neurons is found more laterally than the Tbr1-labeled cluster (red and green arrows, respectively), with the former residing primarily in the interposed and dentate nuclei **(D,D’,F,F’)**, while the latter residing in the fastigial nucleus **(E,E’)**. Panels **(D–F’)** show boxed areas in **(C)** in higher magnification. **(G)** Schematic diagrams illustrating the migration patterns of Pou3f1^+^ (red) and Tbr1^+^ clusters (green) during cerebellar development. DN, dentate nucleus; D, dorsal; EGL, external germinal layer; FN, fastigial nucleus; IN, interposed nucleus; L, lateral; M, medial; NTZ, nuclear transitory zone; RL, rhombic lip; V, ventral; VZ, ventricular zone. Scale bars, 100 μm.

A limited number of other markers of the developing CN neurons have to date been identified. One, Tbr1, labels glutamatergic CN neurons at E13.5 in the NTZ that are destined to populate the fastigial nucleus, and its expression persists into adulthood in these neurons (Fink et al., 2006; Yeung et al., 2016). It is also known that Tbr1 acts downstream of Atoh1 and Pax6, as Tbr1 is not expressed in either the *Atoh1*- or the *Pax6*-null mutants (Fink et al., 2006; Yeung et al., 2016). Thus, we sought to determine how the Pou3f1^+^ cells map onto the Tbr1^+^ fastigial CN neurons. To assess the relationship between Pou3f1^+^ and Tbr1^+^ CN neurons, we performed double labeling of Pou3f1 and Tbr1 between E13.5 and P6. At all timepoints examined, Pou3f1 and Tbr1 are expressed in two non-overlapping populations of cells. In particular, at E13.5, Pou3f1-expressing cells are localized more medially compared to those expressing Tbr1 (Figure 6A). Then, at E15.5, even though Pou3f1^+^ CN neurons are found in the same medial-lateral plane as Tbr1^+^ CN neurons (Figure 6B), it was seen that Pou3f1^+^ CN neurons tend to cluster more ventrally compared to Tbr1^+^ population. At P6 (Figure 6C), Pou3f1^+^ CN neurons populate the interposed and dentate nuclei (Figures 6D,D’,F,F’), lateral to the Tbr1^+^ cells that populate the fastigial nucleus (Figures 6E,E’).

The non-overlapping expression pattern between Pou3f1 and Tbr1 demonstrates that Pou3f1 defines a particular subtype of glutamatergic CN neurons that express neither Pax6 nor Tbr1, and selectively settle in the interposed and dentate nuclei. Moreover, by comparing the spatiotemporal pattern of Pou3f1 expression with that of Tbr1 expression, it is evident that as the cerebellum matures, Pou3f1^+^ CN neurons undergo a medial to lateral migration (Figure 6G; red), while Tbr1^+^ CN neurons exhibit a lateral to medial migration (Figure 6G; green).

### Pou3f1 Co-localizes With Two Other Markers of CN Neurons, Brn2, and Irx3

Brn2 and Irx3 have been previously reported to be expressed in CN neurons. To better delineate the molecular signatures of Pou3f1-expressing CN neurons, we explored the possible co-localization of Pou3f1 with these two transcription factors.

**Figure 7:**
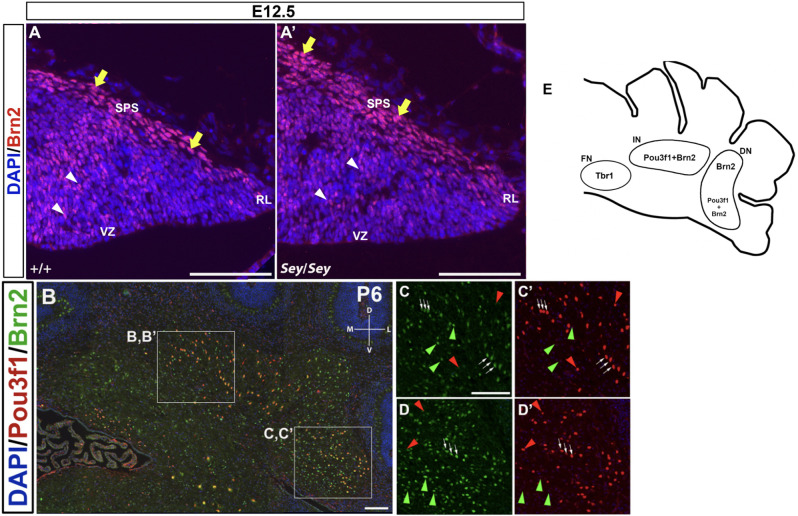
The expression pattern of Brn2 at early (E12.5) and late (P6) timepoints and its relationship to Pou3f1. **(A)** In the E12.5 wild-type cerebellum, Brn2-expressing cells are present along the subpial stream (SPS) (yellow arrows) and in the cerebellar parenchyma (white arrowheads). **(A’)** In the E12.5 *Sey* mutant cerebellum, Brn2 expression recapitulates that in the wild-type counterpart. **(B)** Co-expression of Pou3f1 and Brn2 is evident in both the interposed (**C,C’**, white arrows) and the dentate nuclei (**D,D’**, white arrows). Approximately 70% of Pou3f1^+^ CN neurons also express Brn2. Red arrowheads indicate cells that are Pou3f1^+^ but Brn2^−^, while green arrowheads indicate cells that are Brn2^+^ but Pou3f1^−^. **(E)** A schematic diagram showing the nuclear distributions of Pou3f1^+^, Tbr1^+^, and Brn2^+^ populations in the P6 cerebellar coronal section. Panels **(A,A’)** show sagittal sections, while **(B–D’)** show coronal sections. DN, dentate nucleus; D, dorsal; FN, fastigial nucleus; IN, interposed nucleus; L, lateral. M, medial; RL, rhombic lip, SPS, subpial stream, V, ventral; VZ, ventricular zone. Scale bars, 100 μm.

Brn2, also known as Pou3f2, has been previously described to label CN neurons in the interposed and dentate nuclei (Fink et al., 2006). At E12.5, Brn2^+^ cells are seen along the SPS (Figure 7A), analogous to those expressing Pou3f1. In addition, similar to Pou3f1 expression, Brn2 expression persists in the *Sey* mutant (Figure 7A’), consistent with the notion that the interposed and dentate CN neurons primarily do not depend on Pax6 for their survival.

In co-labeling experiments in the P6 cerebellum, approximately 70% of Pou3f1^+^ CN neurons are found to co-express Brn2 in the interposed and the dentate nuclei (Figures 7B,C,C’,D,D’; white arrows). The presence of Pou3f1-expressing cells that are not labeled by Brn2 demonstrates that Brn2 delineates a subpopulation within the Pou3f1^+^ cohort (Figures 7C,C’,D,D’; red arrowheads). There is also a cohort of Brn2^+^/Pou3f1^−^ cells in the interposed and dentate nuclei (Figures 7C,C’,D,D’; green arrowheads). The small cluster of Brn2^+^ cells close to the fastigial nucleus most likely represents the dorsolateral protuberance described previously (Fink et al., 2006). The nuclear distributions of Pou3f1^+^, Tbr1^+^, and Brn2^+^ populations in the P6 cerebellar coronal section are depicted in Figure 7E.

**Figure 8:**
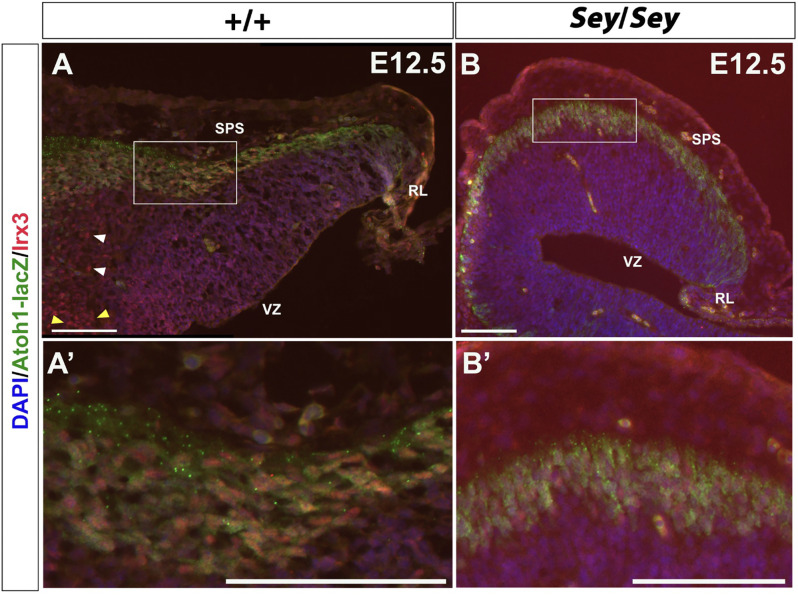
Irx3-expressing cells along the subpial stream (SPS) are derived from Atoh1^+^ progenitors and persist in the absence of Pax6. **(A)** In the E12.5 cerebellum, Irx3^+^ cells are present in not only the ventricular zone (VZ) (yellow arrowheads) and cerebellar parenchyma (white arrowheads), but also in the SPS. **(A’)** Higher magnification image of the boxed region in **(A)**. **(B)** Similar to Pou3f1 and Brn2, Irx3 expression persists in the *Sey* mutant cerebellum. **(B’)** Higher magnification image of the boxed region in **(B)**. The cerebellar sections shown are sagittal sections. RL, rhombic lip; SPS, subpial stream; VZ, ventricular zone. Scale bars, 100 μm.

Irx3 has been shown to label CN neurons emerging from the VZ and is thus described as a putative GABAergic marker (Morales and Hatten, 2006). While Irx3 is expressed in the progenitor cells of the VZ at E11.5 (Morales and Hatten, 2006), at E12.5, IF also reveals the presence of Irx3 expression along the SPS (Figures 8A,A’). Moreover, at E12.5, Irx3^+^ cells along the SPS co-express *Atoh1-LacZ*, which labels the RL-derived progenitor cells that once express *Atoh1* (Figures 8A,A’). These findings indicate that Irx3 in fact also labels future glutamatergic CN neurons. Furthermore, in the E12.5 *Sey* cerebellum, Irx3 expression persists (Figures 8B,B’), similar to Pou3f1 and Brn2 expression.

**Figure 9:**
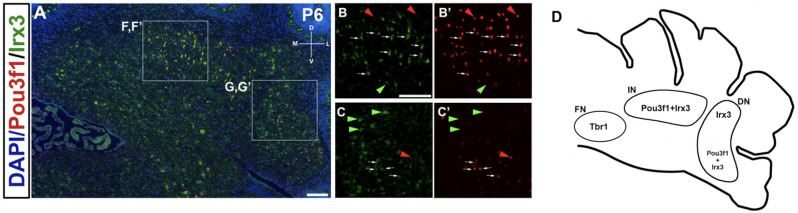
The expression pattern of Irx3 in the P6 cerebellum and its relationship to Pou3f1. **(A)** Co-expression of Pou3f1 and Irx3 is evident predominantly in the interposed nucleus (**B,B’**, white arrows), with minor co-expression in the dentate nucleus (**C,C’**, white arrows). Approximately 55% of Pou3f1^+^ CN neurons also express Irx3. Red arrowheads indicate cells that are Pou3f1^+^ but Irx3^−^, while green arrowheads indicate cells that are Irx3^+^ but Pou3f1^−^. **(D)** A schematic diagram showing the nuclear distributions of Pou3f1^+^, Tbr1^+^, and Irx3^+^ populations in the P6 cerebellar coronal section. DN, dentate nucleus; D, dorsal; FN, fastigial nucleus; IN, interposed nucleus; L, lateral; M, medial; V, ventral. Scale bars, 100 μm.

Co-staining of Pou3f1 and Irx3 in the P6 cerebellum reveals co-localization between these two transcription factors primarily in the interposed nucleus, with minor co-expression in the dentate nucleus (Figure 9A). More than half (~55%) of Pou3f1^+^ CN neurons also express Irx3 (Figures 9B,B’,C,C’, white arrows). As there are also Pou3f1-labeled cells that do not express Irx3, our findings suggest that Irx3 further defines the Pou3f1^+^ population (Figures 9B,B’,C,C’, red arrowheads). Conversely, the Irx3^+^/Pou3f1^−^ cells may well be the GABAergic neurons described previously (Morales and Hatten, 2006; Figures 9B,B’,C,C’, green arrowheads), including the small cluster of Irx3^+^/Pou3f1^−^ cells in the fastigial nucleus. The nuclear distributions of Pou3f1^+^, Tbr1^+^, and Irx3^+^ populations in the P6 cerebellar coronal section are depicted in Figure 9D.

## Discussion

The present study demonstrates that Pou3f1 is a novel marker for glutamatergic CN neurons that settle in the interposed and dentate nuclei. Pou3f1-expressing cells require Atoh1. However, a large portion of cells labeled by Pou3f1 is characterized by the independence from Pax6 for their survival. In addition, the Pou3f1^+^ population does not express Tbr1, a marker of the Pax6^+^ cohort, but instead expresses Brn2 and Irx3. Our results provide further evidence that the glutamatergic CN population is a molecularly heterogeneous population, whose development features the expression of different sets of transcription factors.

### A Molecular Hand-Off Between Pou3f1 and Atoh1 During Glutamatergic CN Neuron Development

Our examination of Pou3f1 and Atoh1 expression in the E12.5 cerebellum suggests a molecular hand-off between Pou3f1 and Atoh1 as cells migrate out of the RL. This process of a switch in gene expression is highlighted by the three regions of the SPS in a posterior to anterior direction: (1) progenitor cells that only express Atoh1 in the RL; (2) cells that co-express the two transcription factors in the SPS proximal to the RL; and (3) cells that are only Pou3f1^+^ in the distal region of the SPS. The downregulation of Atoh1 and the upregulation of Pou3f1 may be a defining point in the development of glutamatergic CN progenitor cells that emerge from the RL. Specifically, the degree of Pou3f1 expression indicates the developmental state of glutamatergic CN neurons: (1) the newly generated CN neuron progenitors are Atoh1^+^ only; (2) followed by cells whose lineage is starting to be restricted that co-express Pou3f1 and Atoh1, while; and (3) the Pou3f1-only glutamatergic CN neurons are the most mature form.

The molecular hand-off between Pou3f1 and Atoh1 may be mediated by negative feedback regulation. Analogous to the regulatory model that governs cerebellar granule cell development, we suspect that, as cells leave the RL, Pou3f1 acts as a negative regulator of Atoh1 expression, much like how NeuroD1 suppresses Atoh1 expression in the granule cells (Pan et al., 2009). As Atoh1 is involved in cell proliferation, by thwarting Atoh1 expression, Pou3f1 then initiates glutamatergic CN neurons to differentiate as they exit the SPS. In fact, the RL presents one of the best models of temporal patterning of cell fate in the CNS, as glutamatergic CN neurons, granule cells, and UBC are all derived from the RL along a strict temporal sequence. However, the molecular patterning specifically within the glutamatergic CN population remains elusive.

### Distinct CN Neuron Populations Are Specified by the Differential Expression of Pou3f1 and Pax6

**Figure 10:**
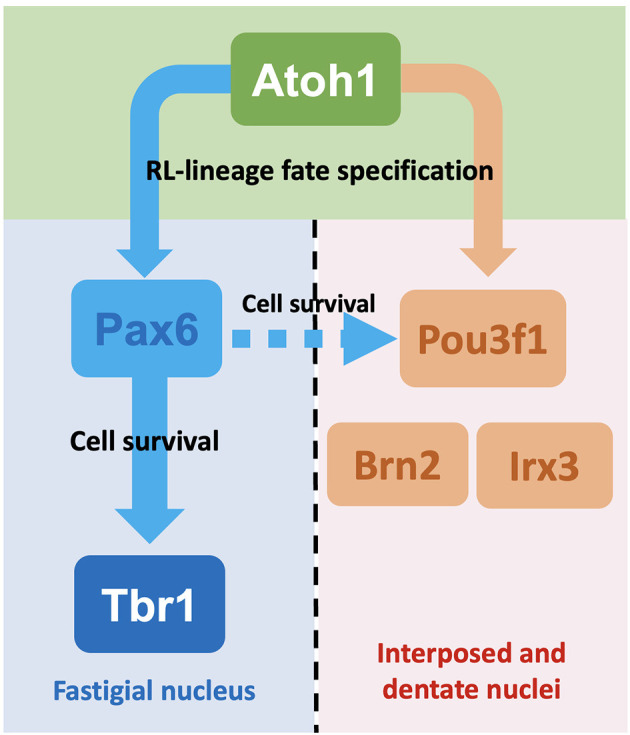
Summaryschematic showing the major findings of this article. Following the expression of Atoh1, glutamatergic CN neurons undergo specification into either the fastigial/medial CN neurons or the interposed and dentate/lateral CN neurons. This process of specification depends on the expression of disparate sets of transcription factors. For glutamatergic CN neurons destined to settle in the fastigial nucleus, these cells subsequently express Pax6 then Tbr1. On the other hand, those destined to settle in the interposed and dentate nuclei express Pou3f1, a subset of which also expresses Brn2 or Irx3. Although different sets of molecules are expressed, the two cohorts of glutamatergic CN neurons also demonstrate cross-talk, as some Pou3f1^+^ cells do require Pax6 for their survival, as indicated by the dashed arrow.

The molecular signatures of the interposed and dentate CN neurons as defined by the expression of Pou3f1, Brn2, and Irx3 diverge from the Pax6- and Tbr1-expressing fastigial CN neurons following Atoh1 expression. This is supported by our observations that Pou3f1 and Pax6 label different clusters of glutamatergic CN neurons during cerebellar development, and that Tbr1, the downstream molecule in the Pax6 program, does not co-label with Pou3f1 in the cerebellum. Interestingly, however, there also appears to be cross-talk between the two CN neuron populations, as the survival of some Pou3f1^+^ cells requires the expression of Pax6. As Pax6’s cell extrinsic effects on the development of nearby cells have been demonstrated previously (Yeung et al., 2016), a model can be proposed here, whereby the Pou3f1^+^ interposed CN neurons, being closest to the Pax6^+^fastigial CN neurons, depend on Pax6 for survival. On the other hand, the Pou3f1^+^ dentate CN neurons, being furthest away from the Pax6 gradient, do not require Pax6 for survival (Figure 10).

Our current findings indicate that the identity of CN subtypes (i.e., fastigial vs. interposed/dentate) is specified during early CN development. One question that remains unknown is how and what regulates the cell fates of the Atoh1^+^ CN progenitors. In the hindbrain, Pax6 has been shown to regulate the cell fates of progenitors, by patterning the Atoh1 and Ngn1 expression domains in the lower RL that give rise to the mossy and climbing fibers, respectively (Landsberg et al., 2005). With the loss of Pax6, the Ngn1 domain expands and the Atoh1 domain diminishes, and that leads to an increase in climbing fibers at the expense of mossy fibers (Landsberg et al., 2005). Similar roles of Pax6 in cell fate specification have been found in the telencephalon (Toresson et al., 2000). In the context of cerebellar development, however, our examination of CN populations in the Sey mutant has not seen an expansion of the Pou3f1-expressing CN population at the expense of the Pax6/Tbr1 population. Thus, the pleiotropic effects of Pax6 in different brain regions remain one of the more fascinating questions in developmental neuroscience, and a topic that invites future studies.

### Relationship Between Pou3f1 and Pax6 Expression and the Migration Patterns of Glutamatergic CN Neurons

Importantly, our results demonstrate that the expression of Pou3f1 vs. Pax6 seems to be a point of divergence that is pertinent to the future colonization of the different cerebellar nuclei by glutamatergic neurons. This notion is supported by our observation of the disparate migration patterns undergone by the Tbr1^+^ fastigial cells and the Pou3f1^+^ interposed/dentate cells, in which the former undergoes a lateral to medial migration during cerebellar development, whereas the latter undergoes a medial to lateral migration. As it has been shown that the CN neurons of the interposed and dentate nuclei are born earlier than those of the fastigial nucleus (Green and Wingate, 2014), we have uncovered here an interesting correlation between migration patterns and birthdates, whereby medial to lateral migration corresponds to early-born CN neurons, while lateral to medial migration corresponds to later-born CN neurons. This finding is supported by recent single-cell work by Khouri-Farah et al. (2022), which identifies Pou3f1 as a marker of the early-born glutamatergic CN neurons, and Pax6 as a marker of the later-born glutamatergic CN neurons.

### Pou3f1 as a Molecular Co-player in the Specification of the Interposed and Dentate CN Neurons

The developmental progression of glutamatergic CN neurons that belong to the fastigial nucleus features the sequential expression of transcription factors Atoh1→Pax6→Tbr1, based upon gene expression and knockout studies (Wang et al., 2005; Fink et al., 2006; Yeung et al., 2016). However, our present findings point to a distinct set of transcription factors involving Pou3f1, Brn2, and Irx3 that play a role in the development of the interposed and dentate CN neurons.

To explore other molecular phenotypes of the Pou3f1-expressing glutamatergic CN neurons, we examined the expression of Brn2 and Irx3, two previously identified markers of CN neurons. We discovered that Brn2 and Irx3 are also markers for the subset of glutamatergic CN neurons delineated by Pou3f1 expression. In the P6 cerebellum, we observed robust co-expression both between Pou3f1 and Brn2 and between Pou3f1 and Irx3. These findings suggest that Pou3f1, Brn2, and Irx3 are all molecular players implicated in the development of a particular subtype of glutamatergic CN neurons. This idea is bolstered by the observation that both Brn2 and Irx3 remain expressed along the SPS in the E12.5 *Sey* mutant cerebellum, similar to Pou3f1, thus indicating that the expression of Brn2 and Irx3 is also independent of the program characterized by the expression of Pax6 and Tbr1.

However, in addition to the Pou3f1/Brn2 and Pou3f1/Irx3 double-labeled populations in the P6 cerebellum, the presence of cells singly labeled by Pou3f1, Brn2, or Irx3 hints that these three molecules may play a role in the development of more than one subtype of glutamatergic CN neurons. Such a possibility is demonstrated in the lateral nucleus, in which the ventral portion is positive for Pou3f1, Brn2, and Irx3 expression, whereas the dorsal portion is positive for either Brn2 or Irx3 expression. This pattern not only reveals that the lateral nucleus can be further characterized into molecularly distinct subnuclei, but also sheds light on the novel idea that different subpopulations of glutamatergic CN neurons are delineated by the combinatorial expression of Pou3f1, Brn2, and Irx3. This molecular diversity of cell types making up the cerebellar nuclei is consistent with recently published single-nucleus transcriptomic data (Kebschull et al., 2020) in which RL-derived excitatory CN neurons were classified into 15 distinct cell types.

The Brn2^+^ and Irx3^+^ cells that do not express Pou3f1, as detailed above, may represent another subtype of CN neurons. Evidence supports the idea that this subtype may be the GABAergic population. Recent single-cell RNA sequencing (scRNA-seq) data (Vladoiu et al., 2019) reported that Brn2 and Irx3 expression is found in both glutamatergic and GABAergic cell types. This would support the notion that within the Brn2^+^ and Irx3^+^ populations, those that are Pou3f1^−^ are GABAergic, while those co-expressing Pou3f1 are glutamatergic. It will be interesting for future studies to unravel the molecular mechanisms driving the non-exclusive expression of Brn2 and Irx3 in both the glutamatergic and GABAergic CN populations.

### Correlating the Molecular Heterogeneity of the Cerebellar Nuclear Progenitor Populations With Their Eventual Functions

The CN neuron population consists of a heterogeneous population of cells: the excitatory glutamatergic CN neurons, as well as the inhibitory GABAergic and glycinergic CN neurons (Chen and Hillman, 1993). CN neurons can be further divided into subgroups based on their mediolateral placement within the cerebellar white matter, as well as their transcriptomic profiles (Kebschull et al., 2020). While it is clear that excitatory and inhibitory CN neurons arise from RL and VZ, respectively, how do the cells that arise from the same germinal zones acquire different subtype identities remains elusive. The present study illustrates that specific subtype identities are acquired early during development. In particular, as glutamatergic CN neuron progenitors leave the RL, the molecular program featuring the expression of Pou3f1, Brn2, Irx3, is first activated and it identifies a population of cells that will colonize the interposed and dentate nuclei (Figure 10). In contrast, the Tbr1^+^ fastigial CN population is generated subsequently following the canonical Atoh1→Pax6→Tbr1 program (Figure 10). Given our current understanding of the anatomical compartmentalization of CN neurons and their projections to other brain regions, the distinct molecular programs come with functional differences. The fastigial CN (Tbr1^+^) neurons project to vestibular and reticular nuclei and are involved in control of head and ocular movements (Ruigrok et al., 2015). On the other hand, the interposed CN neurons project to the red nucleus, which controls sensory and motor processing. The dentate CN neurons project to the thalamus and cerebral cortex (Ruigrok et al., 2015), which play key roles in complex sensorimotor coordination and executive functions. Within the interposed and dentate nuclei, our present work reveals subpopulations characterized by Pou3f1 expression that are further defined by the combination of either Irx3 or Brn2 expression. As Purkinje cells that innervate the CN neurons are also compartmentalized by differential gene expression (e.g., Aldolase C/Zerbin II; Brochu et al., 1990), it will be interesting to know whether there is a correlation in molecularly defined subtype identities between CN neurons and Purkinje cells.

## Data Availability Statement

The datasets presented in this study can be found in online repositories. The names of the repository/repositories and accession number(s) can be found in the article.

## Ethics Statement

The animal study was reviewed and approved by Institutional Animal Care and Use Committee and the Canadian Council on Animal Care at the University of British Columbia.

## Author Contributions

JW, JY, and DG designed research and wrote the article. JW, JY, and MR-B performed research. S-RW and HZ provided the Atoh1 knockout mouse. All authors contributed to the article and approved the submitted version.

## Conflict of Interest

The authors declare that the research was conducted in the absence of any commercial or financial relationships that could be construed as a potential conflict of interest.

## Publisher’s Note

All claims expressed in this article are solely those of the authors and do not necessarily represent those of their affiliated organizations, or those of the publisher, the editors and the reviewers. Any product that may be evaluated in this article, or claim that may be made by its manufacturer, is not guaranteed or endorsed by the publisher.
